# Imaging Mismatch Repair and Cellular Responses to DNA Damage in *Bacillus subtilis*

**DOI:** 10.3791/1736

**Published:** 2010-02-08

**Authors:** Andrew D. Klocko, Kaleena M. Crafton, Brian W. Walsh, Justin S. Lenhart, Lyle A. Simmons

**Affiliations:** Department of Molecular, Cellular, and Developmental Biology, University of Michigan-Ann Arbor

## Abstract

Both prokaryotes and eukaryotes respond to DNA damage through a complex set of physiological changes. Alterations in gene expression, the redistribution of existing proteins, and the assembly of new protein complexes can be stimulated by a variety of DNA lesions and mismatched DNA base pairs. Fluorescence microscopy has been used as a powerful experimental tool for visualizing and quantifying these and other responses to DNA lesions and to monitor DNA replication status within the complex subcellular architecture of a living cell. Translational fusions between fluorescent reporter proteins and components of the DNA replication and repair machinery have been used to determine the cues that target DNA repair proteins to their cognate lesions *in vivo* and to understand how these proteins are organized within bacterial cells. In addition, transcriptional and translational fusions linked to DNA damage inducible promoters have revealed which cells within a population have activated genotoxic stress responses. In this review, we provide a detailed protocol for using fluorescence microscopy to image the assembly of DNA repair and DNA replication complexes in single bacterial cells. In particular, this work focuses on imaging mismatch repair proteins, homologous recombination, DNA replication and an SOS-inducible protein in *Bacillus subtilis*. All of the procedures described here are easily amenable for imaging protein complexes in a variety of bacterial species.

**Figure:**
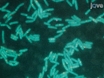


## Protocol

### Growing cultures of cells for microscopy

1. One or two days prior to imaging, prepare the *B. subtilis* strain containing the translational fusion protein you wish to visualize. Trial rounds of steps 1A and 1B will be necessary to determine which growth condition provides the best images of your strain. In most cases, cells should be imaged during exponential growth phase.

For some *B. subtilis* strains (in particular, strains that grow poorly due to the integration of the translational fusion between the gene of interest and GFP at its endogenous locus), initial growth for two days prior to imaging is necessary for high quality images. On the first day, streak the *B. subtilis* strain to be imaged on LB agar with selective antibiotic(s) and incubate overnight at 30°C. On the second day, inoculate 100 μl of 0.85% saline with a single colony and perform three 10-fold serial dilutions on LB agar plates (with selective antibiotics), plating 100 μL of each dilution, followed by incubation overnight at 30°C. On the third day, select the dilution plate with light confluent growth of cells. Light confluent growth on a plate will provide the growth medium with a healthy, exponentially growing starter inoculum to potentially yield excellent imaging results (see step 4). This step is particularly useful for the highest quality membrane imaging with the stain FM4-64.
Other *B. subtilis* strains may simply be applied to selective LB agar medium for single colonies with a sterile stick the day prior to imaging and incubate overnight at 30°C. The following day, cells on this plate will be sufficient to use as an inoculum for imaging (see step 4).*NOTE: For B. subtilis, overnight cultures in liquid medium are not appropriate due to a combination of spore formation and quorum responses, which will cause an extended period of lag phase growth*^1^.

2. On the morning of imaging, place 10 mL of S7_50_^1-3^ medium into a 125 mL sterile Erlenmeyer flask. 

3. Resuspend the cells in up to 2 mL of S7_50_ medium from the LB agar plate with single colonies or the light confluent cells to obtain an inoculum. 

4. Add enough S7_50_ medium-cell inoculum to the Erlenmeyer flask containing 10 mL S7_50_ medium for a culture with an initial optical density measured at 600 nm (OD_600_) of ~0.08 to 0.1. At this step it is important to have at least a 10-fold air to medium ratio. Thus, we use 10 to 12.5 mL of inoculated medium in a 125 mL Erlenmeyer flask. 

5. Grow each culture at 30°C until the culture reaches an OD_600_ ~0.5-0.8. Shaking water baths are preferred with revolutions per minute from 150-200. Cultures challenged with DNA damaging or mismatch inducing agents should be at an early exponential growth phase OD_600_ such that the cells will reach the target OD_600_ with the additional period of growth that is required for treatment (see Table 1) *For example, 2-aminopurine requires one hour of treatment, and therefore a culture treated with 2-aminopurine should be at OD_600_~0.3-0.4, such that the one hour of treatment/growth results in an OD_600_~0.5-0.8, whereby cells are ready for imaging. *

### Sample Preparation

1. Pipette 300 μl of cultured cells into a 1.5 mL microcentrifuge tube.

2. If imaging of cellular membranes is desired, add 1:1000 dilution of FM4-64 membrane stain (Invitrogen) to each sample from a 1 mg/mL stock solution. A titration of FM4-64 may be required to achieve the desired fluorescence signal. A typical titration range is 1:100, 1:1000, and 1:10,000.

3. Allow the samples to sit at room temperature for up to ten minutes or while the slide is being prepared with 1% agarose in 1X Spizizens medium as described below (see step 1 of "slide preparation").

4. If concentration of cells is required, we prefer filtering cells using a 0.2 μm filter with a vacuum apparatus. Cells can then be gently washed from the surface of the filter with PBS, another appropriate buffer or medium prior to visualization.

### Slide Preparation

1. Prepare 1% agarose with 1X Spizizens.Add 5 mL of 10X Spizizens stock to 45 ml ddH2O along with 0.5 g agarose, and microwave to melt. Hot molten agarose is difficult to pipette, and solidified agarose will not enter the pipette tip. The agarose should be equilibrated in a water bath to a temperature of ~55-65°C for it to enter the pipette tip without solidifying within the tip.

2. Draw 20 μl of Spizizens with 1% melted agarose into the pipette tip. Pipette a single drop (approximately 1-2 μL) of the agarose solution into each slide well (we use 15-well slides, see Table 2) to form shallow agarose pads. The agarose will solidify immediately. Slides must also be made immediately prior to application of the cells. If the agarose pads are left at room temperature alone, they can dehydrate within minutes and become unusable. Agarose pads should be centered and fill each well but have minimum height to avoid disrupting the coverslip; if droplets are too high (bubble-like) or if the sample spreads beyond the well boundary, simply swipe the well clean with a Kimwipe and apply a new agarose pad. 

3. Apply the entire 300 μl sample (see step 6) to the slide, distributed on the top of each agarose pad. This amount of cells should be sufficient to cover each well on the slide.

4. Allow the slide loaded with cells to sit at room temperature (23°C) for approximately ten minutes. This step will allow for the cells to settle on the agarose pad becoming immobile and ready for imaging. For specialized needs this temperature may need to be changed, for example, when performing experiments that require a temperature shift^4^.

5. Aspirate away the excess medium from each well without removing the pads themselves.

6. Apply the coverslip to the slide, pressing firmly and evenly, but gently, across the area of the slide. Check to make sure that the cover slip is against the slide and that the cover slip is not raised above the slide.

### Visualization of cells

1. Many different fluorescence microscopes will work well. It is critical to have a 100X oil immersion lens. The Simmons Lab uses:

Olympus BX61 microscope equipped with 1,45 NA TIRFM 100X oil immersion objective lens
Hamamatsu ORCAR^2^ CCD cooled camera
Lumen 200 arc metal (Prior) light source.
For detection of GFP (FITC), filter excitation 460-500 and emission 510-560
For detection of FM4-64 (TRITC), filter excitation 510-560 and emission 572-648.
Images were captured using SlideBook 4.2 (Figure 1)

2. Bring the cells into focus using white light.

3. Capture the image using appropriate filters for each fluorophore. 

### Representative Results

Representative images are shown (Figure 1). GFP foci should be well defined, while FM4-64 staining should be bright and clear ^4-7^. GFP or membrane images can be pseudo-colored with any color, to allow for the highest quality image to be presented without losing any data.


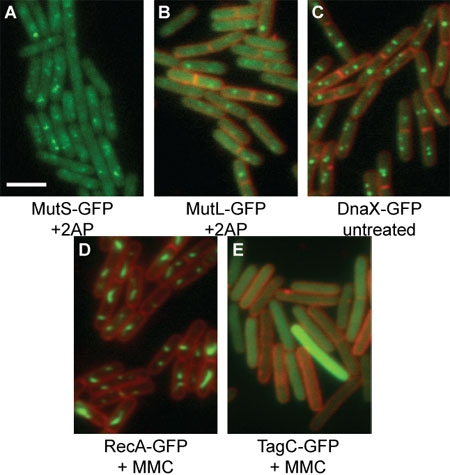
 **Figure 1: Representative GFP images of *B. subtilis*** **translational fusion proteins.** GFP proteins are shown in green, while FM4-64 membrane staining is shown in red. The white scale bar indicates 3 μm. **(A)** MutS-GFP [relevant genotype: *mutS*-*gfp *(*spc*), *amyE*::Pspac*mutL *(*cat*)] in the presence of 600 μg/mL 2-aminopurine and 1 mM IPTG. FM4-64 signal is not presented in order to more clearly show the MutS-GFP foci. **(B)** MutL-GFP [relevant genotype: *mutL::mutL-GFP (spc*)] in the presence of 2-aminopurine. **(C)** DnaX-GFP [relevant genotype: *dnaX::dnaX-GFP (spc*)]. (**D)** RecA-GFP [relevant genotype: *recA::recA-GFP (spc*)] in the presence of 20 ng/mL mitomycin C. **(E)** TagC-GFP [relevant genotype: *tagC::tagC-GFP (spc*)] in the presence of 1 μg/mL mitomycin C. 

## Discussion

Trial and error are required to find exposure conditions for the highest quality images for each strain; we find that 1 millisecond is appropriate for white light images, while exposures of 100 to 2000 ms are appropriate for GFP (FITC) and FM4-64 (TRITC) images. Exposure time will vary depending on the imaging equipment used. We recommend the use of one strain per 15-well microscope slide for the simplest imaging, as pad quality and diffusion of cells from the pad border could complicate strain differentiation if multiple strains are present on the same slide. Image quality directly depends on the quality of the agarose pad where the bacteria are resting. The highest quality images will be captured from agarose pads where bacteria are side by side. Pads that cause the bacterial cells to clump, preventing a monolayer, will produce poor images. Pads that are too thick will produce a high background fluorescent signal, while pads that are dehydrated will not allow for the cells to rest properly. These agarose pad defects will usually result in very poor image quality. If a well is producing poor images, simply move on to the next well. It is ideal to capture between 50 and 200 cells per image. Fluorescence microscopy has provided a wealth of information detailing the cellular cues that direct the assembly of DNA repair and replication proteins into foci *in vivo*. With practice, this technique can be successfully applied to a variety of protein complexes in many bacterial species.
